# Physiological Changes in Women’s Skin During the Menstrual Cycle: A Scoping Review

**DOI:** 10.7759/cureus.75286

**Published:** 2024-12-07

**Authors:** Mai-Linh Nguyen, Sherilyn Nguyen, Nikita Sood, Snigdha Marivada, Alexandra Magaldino, Harvey N Mayrovitz

**Affiliations:** 1 Medical School, Nova Southeastern University Dr. Kiran C. Patel College of Osteopathic Medicine, Davie, USA; 2 Medical Education, Nova Southeastern University Dr. Kiran C. Patel College of Allopathic Medicine, Davie, USA

**Keywords:** menstrual cycle, skin blood flow, skin elasticity, skin physiology, skin sweating, skin temperature, tissue hydration

## Abstract

Multiple physiological changes occur during the menstrual cycle; many are attributed to fluctuations in estrogen, luteinizing hormone, follicle-stimulating hormone, and progesterone. These hormones differentially affect the menstrual cycle's follicular, ovulation, and luteal phases. Skin is one of the organs affected by changes in a woman’s menstrual cycle. However, the understanding of the impact of these composite changes on skin biophysical and physiological parameters is limited. This scoping review was performed to help clarify the extent of physiological changes in the skin during a woman’s menstrual cycle. Skin elasticity, hydration, temperature, blood flow, and sweating were the parameters assessed in this review. Embase, Ovid MEDLINE (Medical Literature Analysis and Retrieval System Online), and Web of Science databases were used to search for peer-reviewed articles in English relating to skin physiological changes during healthy women’s menstrual cycles. The initial search yielded 666 unique articles that met the inclusion criteria. After critical appraisal, further screening produced 192 full texts that resulted in 26 articles that were investigated for skin elasticity, hydration, temperature, blood flow, and sweating during the menstrual cycle. The review clarifies the connection between female reproductive hormone fluctuations, phases of the menstrual cycle, and its subsequent impacts on the skin’s physiological properties. An increase was seen in skin elasticity during ovulation compared to women in the follicular phase. No significant changes were seen in skin hydration across the three menstrual cycle phases. A higher basal skin temperature has been reported during the luteal phase than the follicular phase. A statistically significant increase in skin blood flow was also seen during the mid-luteal phase compared to the follicular phase. Lastly, an increased sweating rate was also observed in the luteal phase compared to the follicular phase for the parameter of sweating. However, higher sweating rates were also reported during the early follicular phase than in the mid-luteal phase. The overall findings of this review highlight how skin physiology varies within the menstrual cycle. This information can be useful in aligning treatment for women with abnormal menstrual cycles or possible maintenance of healthy menstrual flow. This review provides valuable information in a dermatological context to further explore how healthcare providers can apply personalized therapeutic approaches that align with certain phases of a woman’s menstrual cycle, allowing for better skin condition management and improved patient care.

## Introduction and background

The menstrual cycle is associated with significant changes in multiple hormones that have varying physiological effects. The main hormones involved are estrogen, luteinizing hormone (LH), follicle-stimulating hormone (FSH), and progesterone [1]. The three phases of the menstrual cycle (follicular, ovulation, and luteal) are controlled by varying levels of these hormones [2]. FSH upregulates the release of 17-beta-estradiol, the main hormone that carries out the follicular phase [3]. The main purpose of the follicular phase is to recruit and mature ovarian follicles for release during ovulation [4]. At the end of the follicular phase, levels of 17-beta-estradiol are high enough to cause positive feedback that increases FSH and LH, resulting in an LH surge at the start of ovulation. This surge in LH leads to follicle degradation, the release of an oocyte, and the downregulation of 17-beta-estradiol. The degraded follicle forms the corpus luteum, which secretes progesterone, the main hormone of the luteal phase [3]. The corpus luteum is tasked with preparing the endometrium for implantation and does so as long as it is supported by LH. LH relies on human chorionic gonadotropin (hCG) produced through pregnancy, or else it undergoes luteolysis and becomes scar tissue, the corpus albicans [2].

One of the organs affected by these hormonal changes is the skin via alterations in its physiology and physical properties. The impacts of the menstrual cycle on skin physiology depend on changes in the amount of estrogen and progesterone occurring during the cycle's different phases [5]. In turn, many skin parameter changes that occur may depend on the actions of these hormones on skin receptors for estrogen [6], and progesterone [7]. Type I collagen within the reticular dermis and type III collagen within the papillary dermis both increase in response to increased estrogen [7,8], thereby tending to increase skin thickness and improve the structural properties of skin [9]. The structural properties of the skin include protection from environmental damage, thermoregulation, regeneration, and prevention of dehydration. Such structural changes would impact the skin's biophysical properties, including its elasticity and resistance to deformation. Moreover, skin hydration, which depends partly on the ability of lipids within the stratum corneum to hold water, is modulated by estrogen levels [10]. This aspect is expected to result in a variation in transepidermal water loss (TEWL) that might be tracked with the hormone changes through the menstrual cycle. Consistent with this thought is the fact that skin surface lipids are greatest between days 16-20 of the cycle [11]. Multiple other physiological actions of estrogen and progesterone could contribute to menstrual-related biophysical changes such as skin blood flow (SBF) [12].

Reported changes in skin include temperature [13], sweating rate [14], blood flow [5], and mechanical properties [15]. Understanding the physiologic changes in women’s skin during their menstrual cycle can help improve patient outcomes during certain medical treatments such as laser therapy or isotretinoin use [16,17]. Moreover, tracking menstrual cycles and associated symptoms can empower women to recognize and anticipate what and when changes occur in their skin. Knowledge regarding skin changes during the menstrual cycle can also help women better manage their health and prepare them for more informed discussions with their healthcare providers regarding personalized skincare routines and possible medical interventions.

Fluctuations in reproductive hormones play a major role in skin's physiological properties. One example of the broad effect the menstrual cycle has is illustrated by the role of hormones in thermoregulation that impacts skin temperatures and blood flow [6]. Studies have shown an increase in skin temperature and SBF during the luteal phase compared to the follicular phase of the menstrual cycle. In addition, some studies report no overall menstrual cycle-related effects on sweating [18] whereas others report a higher sweating rate in the luteal phase during exposure to heat [19]. Differences in cutaneous vasodilation across the menstrual cycle have been implicated [20]. These and other changes in skin physiology will be further discussed in more detail in this review.

In addition to impacts on skin temperature and sweating features, other skin-related aspects have been the targets of women’s skin investigations. These include the measurement of skin hydration [21], elasticity [22,23], and transepidermal water loss (TEWL) [24]. However, except for two studies done over 10 years ago the menstrual cycle-related physical or physiological changes accompanying these parameters have been little reported [5,21]. 

The purpose of the present review was to identify, characterize, and discuss the range of physiological changes in the skin of healthy women who have a regular menstrual cycle to provide a cohesive overview of the menstrual cycle’s effect on women’s skin.

## Review

A scoping review was chosen as the research modality in order to provide a cohesive summary of the menstrual cycle’s effects on skin and inform future research in this field. This review was conducted in accordance with the Joanna Briggs Institute (JBI) Reviewer Manual [25].

Inclusion and exclusion criteria

To meet inclusion criteria, articles had to be peer reviewed, written in English, involve human subjects, and have included in the article measurements of at least one of the following skin features: elasticity, lipids, blood flow, TEWL, hydration, sweating, and temperature in relation to hormonal fluctuations in the menstrual cycle. Excluded from this review were reviews, abstracts only, studies that did not focus primarily on skin physiology, and studies that did not involve actively menstruating women.

Information sources and search strategy

**Figure 1:**
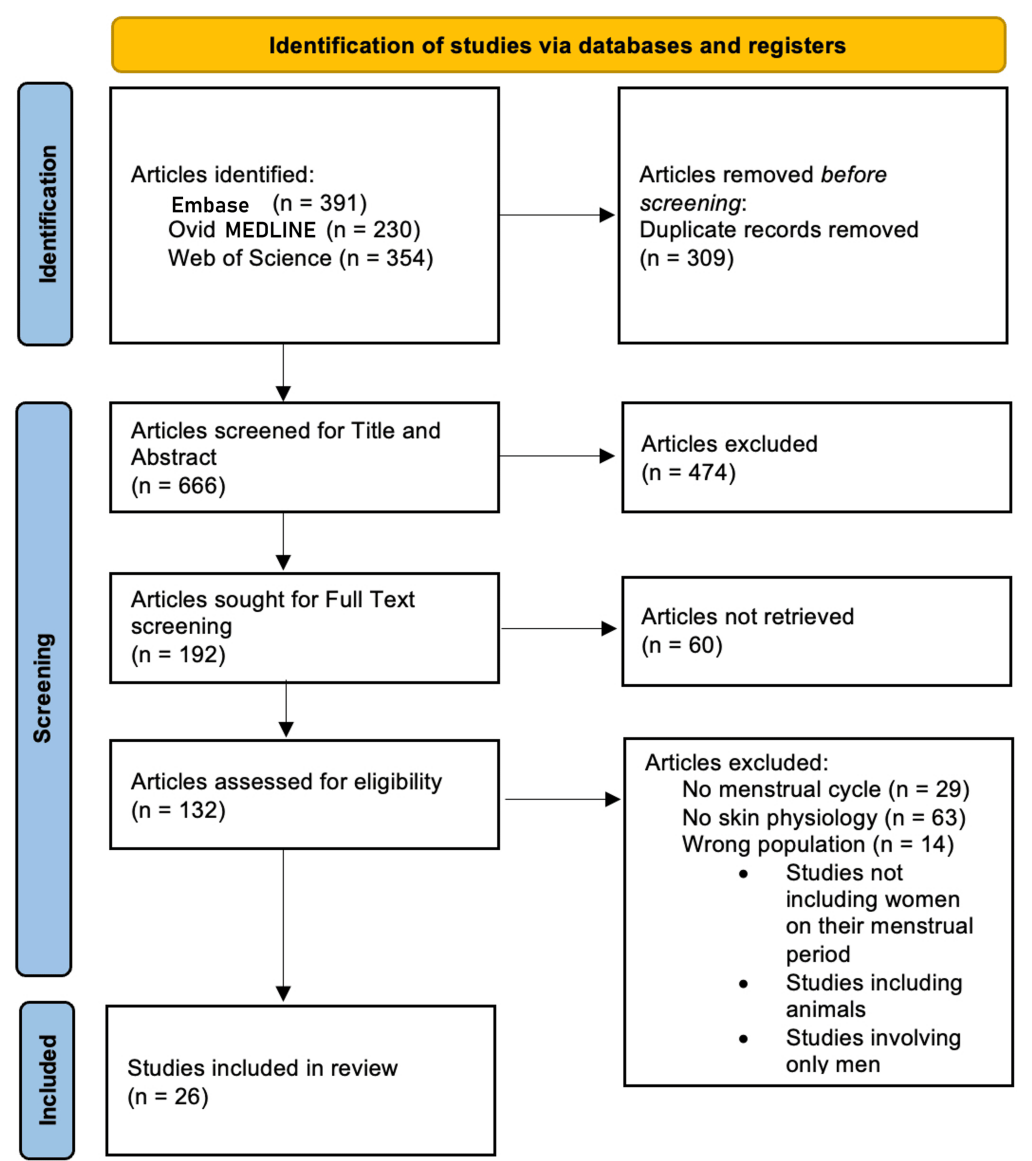
PRISMA diagram showing the search and screening PRISMA: Preferred Reporting Item for Systematic Reviews and Meta-Analyses; MEDLINE: Medical Literature Analysis and Retrieval System Online

Embase, Ovid MEDLINE (Medical Literature Analysis and Retrieval System Online), and Web of Science were the databases searched. All searches were conducted in October 2023. The keywords “skin” and “menstrual cycle” were searched, along with related subject headings. Details of the search string can be found in Appendix 1. The screening and selection process is depicted using the Preferred Reporting Items for Systematic Reviews and Meta-Analyses (PRISMA) flowchart as shown in Figure 1. A total of 975 records were identified from the three databases. Deduplication removed 309 articles, leaving 666 articles to screen. Two reviewers read through titles and abstracts (n= 666) to perform the initial screening process. A third reviewer resolved any conflicts during the screening process. A total of 474 articles were excluded after title and abstract screening for not meeting the previously stated inclusion criteria. Four reviewers read through full text (n=192) and included articles that met the inclusion criteria. A fifth reviewer was added to resolve any conflicts. Sixty articles were not retrieved for full-text screening due to reasons including but not limited to lack of full-length text, discontinued journals, or non-English text.

Selection of sources of evidence and data extraction

Four authors performed full-text screening. Two authors each reviewed half of the full-text articles, while two authors reviewed the remaining half of the full-text articles. To ensure consistency, all authors discussed screening criteria and items for data extraction before conducting the full-text screening. Then all authors systematically assessed the titles, abstracts, and full text for relevant information that warranted inclusion. Discrepancies in study selection and data extraction were discussed with all authors if needed to achieve consensus. A data-charting form to decide on the variables for data extraction was created by two of the authors. Each reviewer independently charted relevant data onto this form throughout the extraction process. 

Critical appraisal

The following data was extracted: title, authors, year of publication, study objective, study design, outcome, key measurements, type of skin physiology studied, and conclusions (Table 1). A thorough critique of all articles after full-text screening was performed using The JBI Appraisal Tools [26]. Included are checklists based on the type of study being critiqued that focus on eliminating biases and assessing the methodology of statistical analyses. The article's overall quality was initially considered by two authors and compared to reach a consensus. Disagreements were consulted and discussed with the remaining authors. Certain aspects of the checklists were prioritized to ensure high-quality data. These include, but were not limited to, assessing the possible risk of bias in a study’s design and data analysis and if there were methods in place to minimize errors during data extraction.

**Table 1:** Characteristics of included studies

Author, Year	Population Size	Skin Parameter
Carpenter and Nunneley, 1988 [27]	8	Temperature
Greenstein et al., 1996 [28]	50	
Lei et al., 2017 [29]	15	
Uchida et al., 2019 [30]	6	
Yanfen et al., 2017 [31]	52	
Matsuda-Nakamura et al., 2015 [32]	13	
Zhang et al., 2020 [33]	9	
Shilaih et al., 2018 [34]	136	
Pivarnik et al., 1992 [35]	9	
Nassar and Smith, 1975 [36]	13	
Charkoudian et al., 1999 [12]	6	Skin blood flow
Bungum et al., 1996 [37]	15	
Inoue et al., 2005 [20]	10	
Kenny et al., 2008 [38]	16	
Kolka and Stephenson, 1997 [39]	5	
Lee et al., 2014 [19]	10	
Martin et al., 2019 [40]	8	
Park and Watanuki, 2005 [41]	20	
Sebzda et al., 2018 [42]	12	
Garcia et al., 2006 [43]	11	Sweating
Kuwahara et al., 2005 [44]	17	
Nose et al., 2020 [45]	28	
Okamoto and Amano, 2021 [46]	20	
Stephenson and Kolka, 1999 [47]	6	
Petrofsky and Lee, 2015 [48]	15	Elasticity
Küçükaydoğan et al., 2020 [49]	10	Tissue water/hydration/moisture

Skin temperature

Ten studies meeting inclusion criteria were evaluated in which a total of 300 women were measured. Study durations ranged from six months to two years with measurements occurring during the follicular and luteal phase. Two studies found that the average skin temperature was significantly lower during the menstrual phase [31,34]. One study reported a statistically significant (p<0.01) skin temperature on the first day of menstruation was 0.18^o^C lower than when measured three days after menstruation [31]. Another study reported a statistically significant (p<0.05) lower skin temperature, measured at the wrist [27]. Average wrist skin temperature was 0.72^o^C lower during the menstrual phase compared to the luteal phase.

Three studies examined skin temperature around ovulation [27,28,36]. One study reported increased temperature during the luteal phase, [27]. Mean skin temperature was determined from the average of values from thermosensors on the chest, arm, thigh, and leg. The study reported a statistically significant increase (p<0.05) in average skin temperature during the luteal phase at 37.6^o^C compared to 37.3^o^C during ovulation and 37.4^o^C during menstruation. The second study measured fingertip skin temperature in women diagnosed with Raynaud’s phenomenon when they were exposed to environmental temperatures ranging from 15^o^C to 35^o^C [28]. The study found that skin temperature was significantly higher (p<0.05) during the menstrual phase compared to the mid-luteal phase at an environmental temperature of 35^o^C [28]. No significant changes were found at the other measured environmental temperatures. The third study reported eight of 13 women with breast skin temperature peaked during the preovulatory period one to two days before ovulation [36]. Four studies found that skin temperature was not affected by the menstrual cycle [29,30,32,36]. These studies measured skin temperature at various sites. One of the studies measured skin temperature at the foot [30] while the other studies averaged skin temperature at the chest, finger, and hallux [32] or chest, arm, calf, and thigh [35].

SBF

A total of nine studies that included 102 women satisfied the inclusion criteria regarding SBF and the menstrual cycle [12,19,20,37-42]. Women who participated in these studies varied in characteristics related to oral contraceptive use, age, and level of physical activity. SBF was measured with laser Doppler flowmetry that reports values in arbitrary units [50-52]. It was measured at varying locations including the thigh, forehead, forearm, and finger. Four out of the nine studies, with a total of 33 subjects, showed a statistically significant increase in SBF during the luteal phase compared to the follicular phase [19,20,39,40]. The reported increases varied by study. In one it was reported as about 50 arbitrary perfusion units, in another, around 80%, in a third by 3.7 mL/100 ml/minute, and in a fourth as 6% of maximum cutaneous vascular conductance [19,20,33,40]. All of these changes were reported as statistically significant. However, in one study the increase was only noted on the back and not on the forehead, forearm, chest, or thigh [20]. Another study found that forearm SBF with aerobic exercise was 3.7 mL/100 ml/min higher during the mid-luteal phase compared to the early follicular phase [39]. To determine this, subjects did leg exercise through a cycle ergometer at 80% aerobic power for about 30 minutes, and forearm blood flow was measured using venous occlusion plethysmography [39]. The other five studies, which included 69 subjects, showed no statistical differences in SBF between the follicular and luteal phases of the menstrual cycle [12,37,38,41,42]. In these studies, SBF was measured at the forearm and leg. These findings collectively highlighted the variability in menstrual cycle-related variations in SBF and the increase during the luteal phase may suggest that hormonal fluctuations during the menstrual cycle do influence it.

Sweating

Five studies, that included a total of 76 healthy women, satisfied the inclusion criteria for the assessment of the effect of the menstrual cycle on sweating [43-47]. Exercise was used as an intervention in all five studies to compare exercise-induced sweat differences during the follicular versus the luteal phase. Parameters used to measure sweating included sweat volume, sweat rate, and sweat output per gland. Two of the five studies that included 37 women found a higher rate of sweating in the follicular phase compared to the luteal phase [44,46]. In the first study, after 30 minutes of cycling exercise, the sweating rate was 0.1 mg/cm^2^/min greater during the follicular phase versus the luteal phase [44]. The other study reported a 50% greater sweating rate in the follicular phase compared to the mid-luteal phase at the forearm and chest sites during exercise [46]. There was also a higher sweat gland output during the follicular compared to the luteal phase in this study. Another study found a higher sweating rate during the luteal phase vs. the follicular phase with values reported as 3.10 ± 0.81 g/m^2^/min vs. 2.80 ± 0.64 with a p-value of p < 0.05 [43]. Only one study, which included 14 women, found that sweat volumes did not differ significantly between follicular and luteal phases with exercise [45]. 

Skin elasticity

Only one study satisfied the inclusion criteria for the assessment of skin elasticity [48]. This study measured the skin elasticity of plantar fascia during the early follicular and ovulation phases. Fifteen healthy women aged 18-35 years were tested once in the follicular phase and once during ovulation. Foot length was measured using a digital caliper under two conditions, one with the person standing on one foot and another while standing on both feet to determine plantar fascia elasticity. Significant increases in foot length were found during ovulation compared to menstruation when standing on two feet (p = 0.03) and on one foot (p = <0.001). In addition, plantar fascia thickness was measured using ultrasound, and a significant increase in plantar fascia thinning was found during ovulation compared to menstruation (p = 0.014). These two changes in measurement, foot length, and plantar fascia thickness, showed an increase in plantar fascia elasticity during the ovulation phase of the menstrual cycle in these healthy women. 

Skin tissue hydration

Only one study satisfied the inclusion criteria for the assessment of skin tissue hydration [49]. This study evaluated 51 females aged 12-45 years. The study measured changes in skin moisture at three phases (early follicular, late follicular, and midluteal). Measurements were done using a device that had four probes to measure pH, sebum, moisture, and erythema. Five sites on the face were measured in each individual. The study found no significant changes in skin moisture across the three phases.

Discussion

A scoping review was conducted to analyze and consolidate evidence surrounding the changes noted as women experienced the luteal, ovulatory, follicular, and menstrual stages within a menstrual cycle. Following the screening and selection process, a total of 26 studies were assessed for the influence of menstrual stages on skin temperature, SBF, sweating, skin elasticity, and tissue hydration. Articles ranging from cohort studies to randomized controlled trials were reviewed for characteristic changes.

Temperature

Skin temperature was reported to undergo several changes throughout the menstrual cycle. In a regular ovulatory cycle, the basal core temperature is normally higher during post-ovulation (luteal) compared to the pre-ovulation (follicular) phase. During ovulation, there is a sudden transition from lower core temperatures to higher core temperatures. These higher temperatures observed in the luteal phase are noted to be present due to the increased levels of progesterone [12,27]. The hypothalamus releases pulsatile pulses of gonadotropin hormone-releasing hormone, activating LH and FSH release by the anterior pituitary, prompting maturation of the follicle and ovulation. Following the formation of the corpus luteum in ovulation, theca and granulosa cells release progesterone in preparation for a possible pregnancy. The dynamic interactions between progesterone and the hypothalamus elevate the basal body temperature [53]. An increase in core temperature during the luteal phase can be attributed to the enhanced heat production leading to increased basal metabolic rate [14]. During the luteal phase, a 5-9% increase in basal metabolic rate and energy expenditure was found. In sequence with the rise of temperature and progesterone post-ovulation, this change carries into the luteal phase, subsequently manipulating the basal metabolic rate and sympathetic activity of the body.

It is important to note that estrogen and progesterone play a critical role in thermoregulatory control. Estrogen counterbalances the influence of progesterone in the latter half of the menstrual cycle, thus aiding in body temperature regulation. The results from this scoping review focused on skin temperature. Two studies reported increased luteal phase skin temperatures of 0.3-0.6^o^C [27,36]. Other studies reported a skin temperature decrease during ovulation. This might be attributed to high levels of estrogen overriding progesterone effects within the first two days of ovulation. Contrastingly, four of the studies included in this review found no significant skin temperature changes among the different phases of the menstrual cycle [29,30,32,36]. Possible explanations for these different findings include study design and environmental factors that influence the results in varying studies. Additionally, these inconsistencies may be attributed to differences in patient variability such as age, body mass index, sensitivity to hormonal changes, and rate of internal metabolic processing. Despite these discrepancies, more than half of the papers researching skin temperature identified a consistent finding aligning with the rise of progesterone during the luteal phase and skin temperature.

SBF

Skin temperature and SBF are interconnected and overlap in their augmentation of internal systems like the vasodilator response. When skin temperature increases, blood vessels dilate and increase SBF. Thus, skin temperature and blood flow work together to influence thermoregulation and monitor the body’s heat exchange. Estrogen and progesterone contribute to this by concentrating this effect in specific areas of the human body. High levels of estrogen and progesterone can increase levels of nitric oxide, a vasodilator, which relaxes blood vessels and reduces vascular resistance [37]. This might explain the reported findings of a higher luteal phase SBF since progesterone levels rise significantly during this phase. Increases in estrogen, which can also cause vasodilation and metabolism, may also be involved. The effects on the skin appear to be somewhat selective, a feature illustrated by a study in which fingertip and forearm skin were compared [37]. There was no change observed in the finger pulp due to thermoregulatory arteriovenous shunts, arteries, and capillaries yet there were changes observed in the forearm, where these structures are absent. Multiple studies also found that SBF in the forearm was higher in the follicular phase compared to the luteal phase [19,20,39,40]. Additionally, local warming of the skin was found to elevate cutaneous vasodilatory response with the influence of estrogen and progesterone.

Sweating

One study found that the sweating rate is usually higher in the luteal phase compared to the follicular phase. The increase in sweating rate in the luteal phase can be attributed to the associated increase in basal core body temperature and elevated progesterone levels. The increased sweating rate observed in the luteal phase may be a compensatory mechanism for the increased basal temperature observed in this phase [43]. However, two studies reported opposite results that indicated higher exercise-induced sweating rates during the early follicular phase compared with the mid-luteal phase [44,46]. These changes might be attributed in part to muscle metaboreceptor activation which is more due to individual response variations than to menstrual effects [46]. Lastly, one study indicated there were no significant sweating rate changes between the two phases [45]. 

Skin Elasticity 

The foot length and plantar fascia elasticity of these women were found to be increased at ovulation in comparison to the women in the menstrual phase [48]. The increase in skin elasticity during ovulation correlates to the surge in estrogen during ovulation. This might be explained by estrogen's ability to promote the production of elastin which can enhance the skin’s elasticity during ovulation [54].

Tissue Hydration 

One study assessed tissue hydration during the menstrual cycle and found there was no statistically significant relationship between tissue hydration and the phases of the menstrual cycle [49]. However, the study mentioned limitations in experimentation relating to observing a single cycle and how every cycle may differ. It further elaborated on the discrepant results found, describing how estrogen typically increases hyaluronic acid levels, a water-retaining molecule, leading to an increase in skin hydration and water-binding capacity. Thus, it was hypothesized that tissue hydration would increase when estrogen levels were the highest during the late follicular phase. However, this was not shown in the study.

Limitations

There are some limitations that need to be considered. This review does not include peer-reviewed articles published after October 2023. There may be selection bias as articles were restricted to only English language publications and searched only using specific databases. The results apply only to women who have healthy cycles and do not include other groups.

## Conclusions

Estrogen and progesterone are influential in the progression of the menstrual cycle, and this review has examined some of their impacts on skin physiology. The relationship between fluctuations in the menstrual cycle phases and some associated skin physiologic changes have been examined with a primary focus on skin temperature, blood flow, and sweating. The relative dearth of reports on skin hydration and skin mechanical property changes during the menstrual phases suggests that more research on these topics is warranted. Knowledge of these variations with the menstrual cycle can be useful in aligning treatment for women with abnormal menstrual cycles or possible maintenance of healthy menstrual flow. Such information may also be used in a dermatological context to explore further how healthcare providers can apply personalized therapeutic approaches that align with certain phases of a woman’s menstrual cycle. 
